# Ovarian hyperstimulation syndrome and prophylactic human embryo cryopreservation: analysis of reproductive outcome following thawed embryo transfer

**DOI:** 10.1186/1757-2215-1-7

**Published:** 2008-11-06

**Authors:** Eric Scott Sills, Laura J McLoughlin, Marc G Genton, David J Walsh, Graham D Coull, Anthony PH Walsh

**Affiliations:** 1 Sims International Fertility Clinic/The Sims Institute, Dublin, Ireland; 2 Faculty of Medical Sciences, University of Newcastle, Newcastle-upon-Tyne, UK; 3 Department of Econometrics, University of Geneva, Geneva, Switzerland

## Abstract

**Objective:**

To review utilisation of elective embryo cryopreservation in the expectant management of patients at risk for developing ovarian hyperstimulation syndrome (OHSS), and report on reproductive outcome following transfer of thawed embryos.

**Materials and methods:**

Medical records were reviewed for patients undergoing IVF from 2000–2008 to identify cases at risk for OHSS where cryopreservation was electively performed on all embryos at the 2 *pn *stage. Patient age, total number of oocytes retrieved, number of 2 pn embryos cryopreserved, interval between retrieval and thaw/transfer, number (and developmental stage) of embryos transferred (ET), and delivery rate after IVF were recorded for all patients.

**Results:**

From a total of 2892 IVF cycles undertaken during the study period, 51 IVF cases (1.8%) were noted where follicle number exceeded 20 and pelvic fluid collection was present. Elective embryo freeze was performed as OHSS prophylaxis in each instance. Mean (± SD) age of these patients was 32 ± 3.8 yrs. Average number of oocytes retrieved in this group was 23 ± 8.7, which after fertilisation yielded an average of 14 ± 5.7 embryos cryopreserved per patient. Thaw and ET was performed an average of 115 ± 65 d (range 30–377 d) after oocyte retrieval with a mean of 2 ± 0.6 embryos transferred. Grow-out to blastocyst stage was achieved in 88.2% of cases. Delivery/livebirth rate was 33.3% per initiated cycle and 43.6% per transfer. Non-transferred blastocysts remained in cryostorage for 24 of 51 patients (46.1%) after ET, with an average of 3 ± 3 blastocysts refrozen per patient.

**Conclusion:**

OHSS prophylaxis was used in 1.8% of IVF cycles at this institution; no serious OHSS complications were encountered during the study period. Management based on elective 2 *pn *embryo cryopreservation with subsequent thaw and grow-out to blastocyst stage for transfer did not appear to compromise embryo viability or overall reproductive outcome. For these patients, immediate elective embryo cryopreservation and delay of ET by as little as 30 d allowed for satisfactory conclusion of the IVF sequence, yielding a livebirth-delivery rate (per ET) >40%.

## Introduction

Ovarian hyperstimulation syndrome (OHSS) is the most serious consequence of ovulation induction and in vitro fertilisation (IVF), potentially resulting in death in its extreme manifestation [1]. How best to manage this condition has been the subject of considerable study, with primary emphasis on risk recognition before commencing the IVF stimulation sequence [2,3]. The exact etiology of OHSS remains unknown. Since pregnancy can worsen OHSS, embryo transfer is sometimes intentionally postponed by electively freezing embryos until symptoms have resolved and the clinical picture improves [1]. In this study, data collected at one IVF referral centre during a nine-year period were used to assess a conservative strategy for OHSS prophylaxis and to investigate how empiric embryo cryopreservation and delayed transfer might impact reproductive outcome.

## Materials and methods

### Patient selection and study design

Patient records for all ovulation induction performed at the Sims International Fertility Clinic were retrospectively reviewed for the period 2000–2008, including all IVF patients (*n *= 2892). No case of OHSS was diagnosed in patients undergoing gonadotropin treatment for IVF. In this population, OHSS prophylaxis using elective embryo cryopreservation was instituted when the number of follicles with mean diameter ≥ 15 mm exceeded 20 [1] and when an intraperitoneal pelvic fluid collection was present measuring >5 cm (in any diameter) on transvaginal ultrasound. Serum oestradiol measurements were not obtained for every patient for the entire duration of the study period, so this parameter was not included for analysis.

All IVF patients included for study received a focused physical examination, and saline infusion sonogram, which were normal before initiating gonadotropin therapy. Controlled ovarian hyperstimulation regimens were developed from factors including historical response to medications, patient age, BMI and ovarian reserve assessment. Pituitary downregulation was achieved with oral contraceptives and GnRH agonist, followed by daily administration of gonadotropins (daily dose ≤ 150 IU/d) with periodic monitoring as previously described [4]. Treatment continued until adequate ovarian response was attained, defined as at least three follicles with mean diameter ≥ 17 mm. Transvaginal sonogram-guided oocyte retrieval was accomplished 36 h after subcutaneous administration of 10,000 IU hCG. Immediately after retrieval oocyte-cumulus complexes were placed into Universal IVF medium (MediCult; Jyllinge, Denmark), with insemination (including ICSI) also carried out using this reagent under washed liquid paraffin oil (MediCult, Denmark). Fertilisation was assessed after 16–18 h and was considered normal when two distinct pronuclei were noted.

**Figure 1:**
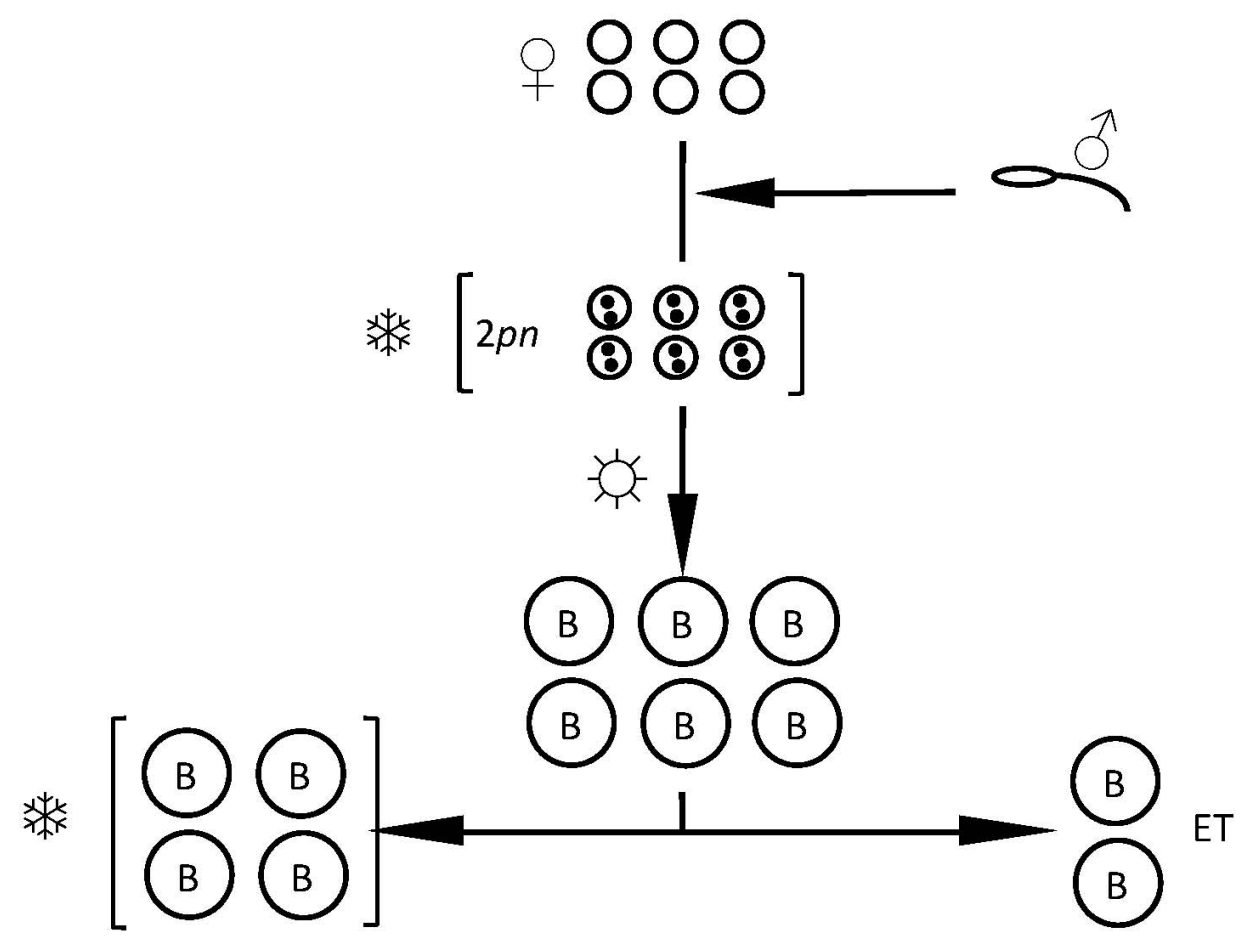
Schematic for prophylactic embryo cryopreservation at the 2pn stage, followed by extended culture to blastocyst stage (B) and subsequent transfer (ET). Non-transferred blastocysts are re-frozen for subsequent use (lower left).

For patients considered at risk for OHSS (based on criteria outlined above), extensive and immediate counselling was provided during the IVF cycle to review the potentially grave risks associated with fresh embryo transfer (as originally planned at cycle initiation). Since fresh transfer was not regarded as safe when OHSS might develop, alternate options of cycle cancellation and elective embryo cryopreservation were carefully outlined. While complete cycle cancellation was uniformly offered, no patients at risk for OHSS elected to do this during the study interval. Consequently, these cases were managed via elective embryo freeze (see Figure 1). Once this "freeze all" decision was made, this was documented and communicated to embryology staff. There were no additional OHSS cases that developed after embryo transfer who were not previously recognised during follicular recruitment and ovulation induction with gonadotropins.

### Embryo cryopreservation sequence

Following confirmation of normal fertilisation by the presence of two distinct pronuclei, embryos were placed in cryoprotectant (Embryo Freezing Pack, MediCult, Denmark) at room temperature and cooled to -7°C at a rate of 2°C/min. Manual seeding followed after 5 min, then the embryos were cooled from -7°C to -30°C at a rate of 0.3°C/min. The final rapid cooling step brought the embryos from -30°C to -190°C at 50 C/min; they were next transferred to liquid N_2 _for long-term storage and maintained at -196°C.

### Thaw, culture & transfer protocols

2 *pn *embryos were removed from liquid N_2 _storage and kept at room temperature ×30 sec before being placed in H_2_O bath at 30°C for 1 min. Embryos were placed in 1,2 propanediol/sucrose-based thaw media (Embryo Thawing Pack, MediCult, Denmark) at room temperature for a total of 20 min. Culture was maintained to day five in microdrops of BlastAssist media I and II (MediCult, Denmark) under washed paraffin oil in a 5%CO_2 _+ 5%O_2 _atmosphere at 95% humidity. Embryos were assessed daily for cell number, degree of fragmentation, and compaction. Day five blastocysts selected for *in utero *transfer generally demonstrated a well-defined inner cell mass and highly cellular, expanding trophoectoderm. Blastocysts were loaded into an ET catheter (K-Soft-5000 Catheter; Cook Medical Inc., Spencer, Indiana USA), and all transfers occurred under direct transabdominal sonogram guidance.

### Secondary freeze for non-transferred blastocysts

Supernumary blastocysts selected for (repeat) cryopreservation were incubated in 5–6% CO_2 _atmosphere at 37°C × 2 h, then placed in cryoprotectant (BlastFreeze, MediCult, Denmark) cooled to -6°C at a rate of 2°C/min. After manual seeding, embryo temperature was taken from -6°C to -40°C at 0.3°C/min. Final rapid cooling of blastocysts from -40°C to -150°C at 35°C/min was followed by transfer to long-term storage in liquid N_2 _at -196°C.

### Outcomes reporting and statistical analysis

Primary endpoints of the study were patient age, total number of oocytes retrieved, number of 2 *pn *embryos cryopreserved, interval between retrieval and thaw/transfer, number (and developmental stage) of embryos transferred (ET), and livebirth-delivery rate. All data were tabulated as mean ± SD. Patients were periodically followed during pregnancy, or contact was established with their delivering obstetrician to determine delivery status. In the event that contact could not be made and delivery status remained unknown, this was also noted in the record.

## Results

A total of 2892 IVF cycles proceeded to oocyte retrieval during the study period. Of these, 51 patients (1.8%) were judged to be at risk for developing OHSS and prophylactic embryo freezing was performed. While none of these patients qualified for fresh embryo transfer according to medical centre policy, there were some patients requesting elective embryo cryopreservation who were not at risk for OHSS. Reasons for empiric embryo cryopreservation in these cases included incidental surgery unrelated to fertility, diagnosis of malignancy, and divorce. Reproductive outcomes for these patients not considered at-risk for OHSS were excluded from the calculation of delivery rates in this study.

The mean (± SD) age of patients at risk for OHSS during the study period was 32 ± 3.8 yrs. Both ovaries were present and morphologically normal at baseline for all patients at risk for OHSS. All patients underwent ultrasound-guided transvaginal oocyte retrieval at our facility without incident; the average number of oocytes retrieved per patient was 23 ± 8.7. After fertilisation either by conventional insemination or ICSI, an average of 14 ± 5.7 2 *pn *embryos were cryopreserved per patient. Embryo cryopreservation was successfully carried out for approximately 61% of the total number of retrieved oocytes in this population.

Over the next 30 d, patients at risk for OHSS were periodically re-evaluated after elective cryopreservation of their embryos to document clinical improvement and resolution of symptoms. When there was no laboratory evidence of haemoconcentration, pelvic fluid collections had cleared, and ovarian quiescence was noted via ultrasound, it was considered safe to resume the IVF treatment sequence: thaw and grow-out to blastocyst stage was carried out. Development to blastocyst stage was achieved for 88.2% of cases, but when the cohort of thawed embryos did not advance to blastocyst stage the most developed embryos were transferred. This affected six cases after thaw, five of whom had day three embryos and one case where a 'mixed transfer' consisting of one morula + one blastocyst was performed. Embryo transfer was performed an average of 115 ± 65 d (range 30–377 d) after oocyte retrieval. In these patients at risk for OHSS, the mean number of embryos transferred was 2 ± 0.6. For 24 of these (46.1%), supernumary blastocysts remained after transfer and were returned to cryostorage.

For patients considered at risk for OHSS where elective 2 *pn *embryo cryopreservation was performed, the live birth delivery rate was 33.3% (17/51) per initiated cycle and 43.6% (17/39) per transfer. No twin or triplet deliveries occurred in this series. Follow-up with patients after delivery identified no long-term OHSS sequela, and there were no malformations or developmental anomalies reported among offspring.

## Discussion

Ovarian hyperstimulation syndrome (OHSS) is a potentially fatal iatrogenic condition resulting from excessive stimulation of the ovaries [5]. The vast majority of OHSS develops in the setting of injectable gonadotrophins used in IVF, although in the absence of proper monitoring oral clomiphene treatment can also result in massive ovarian hyperstimulation necessitating surgical removal of the ovary [6].

According to the World Health Organization (WHO), severe OHSS develops in 0.2–1% of all stimulated ART cycles [7]. Several methods to prevent OHSS have been advocated including elective embryo cryopreservation, using low-dose hCG or GnRH-agonist for triggering oocyte maturation, "coasting" gonadotropin use, and cycle cancellation. To date, no single investigation has compared patient outcome and pregnancy rates among these varied interventions. In the present study, we implemented elective embryo freezing as an OHSS prophylactic measure in 1.8% of our IVF patients, a figure parallel to previous reports of actual OHSS incidence [8,9]. The pathophysiology of OHSS is complex; it likely involves a disruption of inflammatory processes normally evoking ovulation. The characteristic capillary extravasation of OHSS seems to be mediated by interactions of prolactin, prostaglandins, the ovarian prorenin-renin-angiotensin system, vascular endothelial growth factor (VEGF), angiogenin, the kinin-kallikrein system, selectins, von Willebrand factor, and/or endothelin [2]. The altered vascular permeability of OHSS may also be modulated by VE-cadherin [10], an interendothelial adhesion molecule or serum soluble ICAM-1 [11]. Of note, significantly higher IL-18 levels have been detected in the serum and extravascular fluids of patients with severe OHSS compared to non-OHSS controls [12].

When a patent is considered at risk for OHSS at our centre, we do not typically proceed with fresh embryo transfer (ET). During patient counselling, we explain that the strategy of empiric embryo freezing to minimise OHSS risk is not new [1,3,13,14], but has considerably lower risk than proceeding with fresh ET as originally planned. The rationale for delaying ET derives from the intent to delay pregnancy, since hCG increases VEGF which in turn facilitates the endothelial permeability associated with OHSS [15]. One of the first prospective studies to demonstrate the therapeutic benefit of elective early embryo cryopreservation in OHSS patients randomised subjects to undergo either fresh ET or receive delayed ET after cryopreservation (and thaw) of all embryos. No cases of OHSS developed in the setting of elective embryo freeze; pregnancy rates were comparable between the two groups [16].

OHSS risk is not always eliminated by elective freezing of embryos. An earlier review of precautionary cryopreservation of all embryos at the 2 *pn *stage noted that OHSS developed anyway in 27% of cases [13]. Some have speculated that elective embryo freezing may reduce the severity – but not lower the incidence of – symptomatic OHSS [14].

One unexpected finding from the present study was the large variation in time interval between oocyte retrieval and thaw/transfer among patients at risk for OHSS, which ranged from 30 to 377 days. While neither duration of cryostorage nor type of ovulation induction protocol has been found to affect reproductive outcome in IVF [17,18], we nevertheless remain curious why any IVF patient would wait for more than a year to initiate a thaw-transfer sequence – risk of OHSS notwithstanding. It is our belief that such extended (>90 d) delays are a function of patient scheduling preferences rather than medical factors, but the question forms the basis of ongoing research here. As our data show, elective embryo cryopreservation may lead to considerable delay in treatment (and consequently postpones the potential for pregnancy) and many patients at risk for OHSS are understandably discouraged by the prospect of elective embryo freeze. However, patients should be made aware that while live birth delivery rates among IVF patients at risk for OHSS can be impressive [19], the dangers accompanying OHSS are not be underestimated. For example, one series in Ireland recently demonstrated a 4% case fatality rate for the condition [9].

Our study has several limitations which should be acknowledged. We focused more on OHSS prevention rather than development of the condition itself. Serum oestradiol has been used as a marker for OHSS risk for many years [1,2,5,7,9], but this method of screening was not regularly available at our institution throughout the nine-year study period and thus was not part of this analysis. Additionally, the role of paracentesis or albumin/hespan infusion could not be specifically studied in this report because medical records were not electronically searchable for these terms throughout the study period. While 2 *pn *embryos for cryopreservation were produced from approximately 61% of retrieved oocytes in this series, our OHSS cases were not stratified according to ICSI vs. conventional insemination. However, previous research has suggested that pregnancy rates after cryopreservation of 2 *pn *embryos are not impacted by fertilisation method [20]. Our retrospective study also did not have a control group, so it is unknown how many patients might have developed OHSS if a fresh transfer had been performed. However, it is reasonable to conclude that some OHSS cases would have been expected from this high-risk population.

In conclusion, this descriptive study finds conservative application of elective embryo cryopreservation to be a useful component of OHSS prophylaxis. High delivery rates are typical among women at risk of OHSS who undergo elective embryo cryopreservation with deferred thaw/transfer, and our outcomes data support this finding as well. While serum oestradiol determinations can be helpful in OHSS surveillance, this report shows that screening based on clinical parameters can also be effective. Further studies are planned to refine specific factors that might be useful in prediction of OHSS risk, with a view to optimise clinical management of this important and potentially dangerous condition.

## Competing interests

The authors declare that they have no competing interests.

## Authors' contributions

ESS and LJM collected data for the study and prepared the original manuscripts; MGG provided design input and statistical analysis; GDC organised the embryology laboratory components and provided data on gametes and reproductive outcome; DJW and APHW supervised the project and directed the research. All authors approved the final manuscript.
